# Peptide-Based Drug Delivery Systems

**DOI:** 10.3390/medicina57111209

**Published:** 2021-11-05

**Authors:** Dmitriy Berillo, Adilkhan Yeskendir, Zharylkasyn Zharkinbekov, Kamila Raziyeva, Arman Saparov

**Affiliations:** 1 Department of Pharmaceutical and Toxicological Chemistry, Pharmacognosy and Botany School of Pharmacy, Asfendiyarov Kazakh National Medical University, Almaty 050000, Kazakhstan; 2 Department of Medicine, School of Medicine, Nazarbayev University, Nur-Sultan 010000, Kazakhstan; adilkhan.yeskendir@nu.edu.kz (A.Y.); zharylkasyn.zharkinbekov@nu.edu.kz (Z.Z.); kamila.raziyeva@nu.edu.kz (K.R.)

**Keywords:** drug delivery system, drug carriers, peptide-based, cell penetrating peptides, targeted delivery, stimuli responsive, scaffolds

## Abstract

Peptide-based drug delivery systems have many advantages when compared to synthetic systems in that they have better biocompatibility, biochemical and biophysical properties, lack of toxicity, controlled molecular weight via solid phase synthesis and purification. Lysosomes, solid lipid nanoparticles, dendrimers, polymeric micelles can be applied by intravenous administration, however they are of artificial nature and thus may induce side effects and possess lack of ability to penetrate the blood-brain barrier. An analysis of nontoxic drug delivery systems and an establishment of prospective trends in the development of drug delivery systems was needed. This review paper summarizes data, mainly from the past 5 years, devoted to the use of peptide-based carriers for delivery of various toxic drugs, mostly anticancer or drugs with limiting bioavailability. Peptide-based drug delivery platforms are utilized as peptide–drug conjugates, injectable biodegradable particles and depots for delivering small molecule pharmaceutical substances (500 Da) and therapeutic proteins. Controlled drug delivery systems that can effectively deliver anticancer and peptide-based drugs leading to accelerated recovery without significant side effects are discussed. Moreover, cell penetrating peptides and their molecular mechanisms as targeting peptides, as well as stimuli responsive (enzyme-responsive and pH-responsive) peptides and peptide-based self-assembly scaffolds are also reviewed.

## 1. Introduction

A large number of compounds fail to progress through the various stages of preclinical and clinical studies due to a number of reasons, including but not limited to, high cytotoxicity, poor pharmacokinetic rate and inefficient site-specific targeting. Pharmaceutically active substances at physiological conditions should be able to overcome biological obstacles such as albumin binding and aggregation, insolubility, biodegradation/metabolism, the low permeability via vascular endothelial cell layers, rapid excretion by the kidney, inefficient cellular internalization and undesirable immunogenicity [1]. These issues create a narrow therapeutic window and as a consequence, lead to dismal in vivo performance. The development of a novel drug, including all stages of clinical studies, costs about USD 2.2 billion [2]. The use of novel drug delivery systems (DDSs) that improve the properties of a cell membrane’s penetration may provide an opportunity of recycling drug candidates, which have previously demonstrated the above-mentioned disadvantages. Moreover, repeated drug administration raises the cost and, in many cases, causes undesirable side effects. DDSs based on synthetic stimuli responsive copolymers are widely discussed in recent reviews, including thermoresponsive [3,4], pH responsive [5,6,7] and modified natural polymers [8] that improve the stability, the effectiveness of pharmacokinetics and the tolerability of existing substances, concurrently mitigating their off-target toxicity.

A comprehensive review of nanocarriers based on various types of lysosomes, solid lipid nanoparticles, dendrimers, polymeric micelles, virus-based nanoparticles, inorganic and organic/inorganic hybrid nanocarriers discusses advances in the application of carriers with non-immunogenic and biodegradable architecture for having optimal pharmacokinetic and pharmacodynamic parameters of the drugs [9]. Some liposome-based DDSs have been approved by the U.S. Food and Drug Administration (FDA), such as liposomal doxorubicin (Doxil^®^) and liposomal amphotericin B (Ambisome^®^) [10]. A recent review covers standardization parameters of therapeutic peptides and compares the monographs of different Pharmacopeias all over the world, which is an important and obligatory stage for entering the market. Thus, to date, there are about 60 peptides that have reached the market, more than 150 peptides are in active clinical trials and about 260 peptides are currently being tested in humans and over 400 peptides in nonclinical studies [11]. There is also a classification of DDSs based on pharmaceutically active substance delivery to a specific organ, for example, growth factor or cytokine delivery to accelerate and improve tissue repair and regeneration [12,13,14,15,16]. Numerous research studies indicate that biodegradable natural, semi natural, synthetic and hybrid polymers serve as a milestone in the design and development of innovative DDS paradigm, improving the management and healing of damaged tissue, decreasing side effects and improving the pharmacodynamics of the substance [17,18].

On some occasions, extracellular vesicles derived from cells can be utilized for effective delivery of various substances such as proteins, lipids and genetic materials (messenger RNA (mRNA), microRNA, other small non-coding RNA and genomic DNA) to the target cell or organ [19]. The drawback of most of the above-mentioned systems is their poor control of delivery to some target organs or cells, slow pharmacokinetic or release rate, harmful degradation products and unsatisfactory penetration via the blood-brain barrier. There are a number of papers related to peptide-based carriers, which were not discussed in previous review papers, thus filling this gap will generate new ideas for the creation of efficient DDSs and provide such qualities as utilization of a prodrug where a small peptide sequence is covalently conjugated to the active substance disguising its pharmacological activity until it is triggered by a disease-specific agent at the desired site [10]. Encapsulating the drug in a peptide-based DDS dictates the pharmacokinetics and pharmacodynamics through its unique physico-chemical properties and the possibility of the use of an implantable drug-eluting depot. Cooper and colleagues have analyzed peptide-based carriers and the modification of protein sequence to slowing down renal clearance of carriers loaded with drugs. Moreover, they performed a comprehensive analysis of data related to antibody–drug conjugates, which is also a promising trend in targeted therapy [20].

This review highlights the design of drug delivery carriers based on peptides and their advantages compared to synthetic polymer-based DDSs, hybrid particles and composite carriers due to less of a response from the immune system, as well as the absence of toxic products of degradation and the possibilities of modulating the entrance of the carrier via the cell membrane and of designing a specific high affinity sequence that provides efficient binding with the target. Furthermore, the functional efficiency of the peptides and their contribution to the overall therapeutic efficiency of DDSs are reviewed and discussed.

## 2. Cell Penetrating Peptides

### 2.1. Molecular Mechanisms of Delivery of Cell Penetrating Peptides

**Figure 1:**
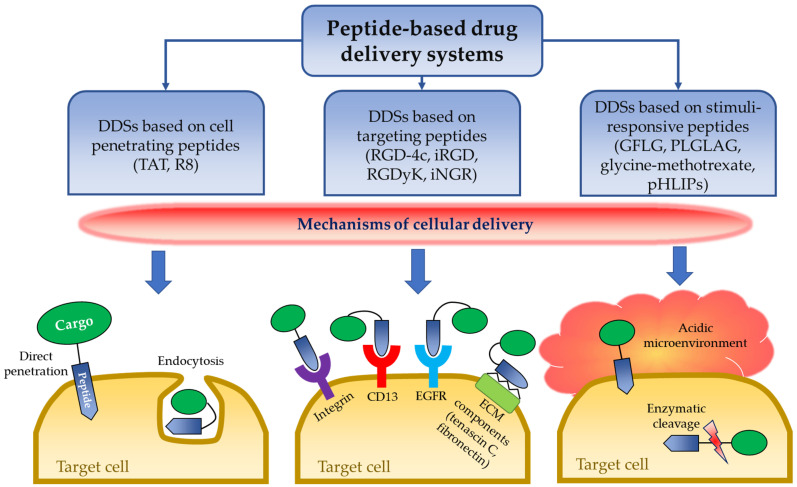
Peptide-based drug delivery systems.

Some peptides can enter the cell without damaging the integrity of the cellular mem-brane and are considered effective and safe DDSs. This class of peptides is usually classified as cell penetrating peptides (CPPs) [21]. CPPs are firstly derived from the α-helical domain of the TAT protein, encoded by the human immunodeficiency virus type 1 (HIV1), and cover residues from 48 to 60 [22]. CPPs are short peptides (less than 30 amino acids), mostly cationic and are able to conjugate therapeutic molecules. Nowadays, CPPs contain more than 1800 different sequences that are validated experimentally [23]. There are two main pathways of cellular uptake for CPPs to penetrate the cell: endocytosis and direct translocation [22]. However, some CPPs can enter the cell through either one of two path-ways (Figure 1). For example, recently published studies demonstrated that a bovine lactoferricin L6 CPP can be internalized by endocytosis, however the addition of polyhistidine peptides to this complex can also allow internalization by a direct membrane trans-location [24,25].

Endocytic internalization of CPPs is an energy dependent process that includes three different pathways: macropinocytosis, caveolae-mediated endocytosis (CvME) and clathrin-mediated endocytosis (CME) [26]. Macropinocytosis is a common nonspecific mechanism of cellular uptake and is activated in response to stimulation by various growth factors, including but not limited to, macrophage colony-stimulating factor-1, the epidermal growth factor (EGFR) and the platelet-derived growth factor [27]. It begins with the rearrangement of the cytoskeleton by actinic cytoskeletal elements leading to the invagination of the cellular membrane and further subsequent formation of a sheet-like pocket and large endocytic vesicles that entrap extracellular fluid and particles in macropinosomes [28]. Nakase and colleagues demonstrated that a single transmembrane domain protein called syndecan is also involved in the initiation of macropinocytosis. After the interaction of peptides with the cellular membrane, multimerization of syndecans and subsequent actin polymerization can occur leading to the macropinocytic uptake of actin-rich pep-tides [29]. Macropinocytosis is the main mechanism for uptake of polyarginine, TAT and NickFect51 [30,31]. In CvME, CPPs with cargo can bind to the cellular membrane and initiate clustering of caveolin-1 proteins via actinic cytoskeletal elements [28]. This leads to the formation of flask-shaped invaginations and further phosphorylation of caveolin-1 resulting in the internalization of the vesicle [26]. This pathway is involved in the uptake of TAT, proline-rich CPPs, PepFect14/DNA conjugate, p18 and p28 azurin fragment [32]. A recent study also revealed that a novel chicken anemia virus derived CPP, designated as CVP1, effectively delivered β-glycosidase, poly (I:C) and plasmid into HCT116 cells via CvME [33]. Finally, CME begins with the initiation of endocytic events by formation of vesicles in phosphatidylinositol 4,5-biphosphate-rich regions of plasma membrane after interaction of peptides with specific cell surface receptors. Thereafter, an adaptor protein binds to phosphatidylinositol 4,5-biphosphate and initiates clathrin assembly to the plasma membrane [34]. This is followed by the invagination of the membrane coated with clathrin towards the cytoplasm leading to the formation of a coated pit. Further transformation of the pit to spherical bud results in the formation of a membrane neck which is cleaved by GTPase called dynamin. Further release of clathrin-coated vesicles rapidly leads to the loss of a clathrin protein coat and their delivery to early endosomes [35,36]. An early endosome matures to a late endosome after its delivery by microtubules to the nucleus. Late endosomes then carry their cargo to organelles and lysosomes with low pH values [28,32]. The involvement of CME in peptide delivery to the cell has been reported for TAT peptide, oligo-arginine and anionic CPPs [32,37].

Direct translocation of peptides is an energy independent process, which occurs at a low temperature and without the participation of receptors. It initially starts with the interaction of positively charged CPPs with the negatively charged membrane components and phospholipid bilayer [38]. Four different pathways were proposed for further CPP internalization by direct translocation: barrel stave model, toroidal pore model, carpet-like model and inverted micelle model [39]. In the barrel stave model, the hydrophilic regions of CPP face parallel to the lipid bilayer surface of the cell membrane. Further, under the influence of high pH and sufficient number of peptides, the surface of the outer cellular membrane changes forming perpendicular pores with hydrophilic residues of the CPP covering the internal environment of the pores [38,39]. The toroidal pore model is characterized by the formation of pores after association of peptides with the polar head groups of lipids inside the cellular membrane. The hydrophilic walls of the toroidal pore are formed by both hydrophilic phospholipid cell membrane and inserted peptides [39]. In the carpet-like model, a sufficient amount of CPPs covers the cell membrane leading to the interaction of hydrophilic groups of peptides with the phospholipid head groups of the membrane without internalization into the hydrophobic core. Subsequent rotation of pep-tides results in the reorganization of the cell membrane leading to the formation of a transient hole in the membrane [39]. Finally, the inverted micelle model is characterized by the formation of micelles between the outer and inner membrane bilayers after interaction of CPPs with the cell membrane. Hexagonal micelles cover CPPs by their hydrophobic surface and it also allows transportation of hydrophilic compounds conjugated to the peptide. Further, release of peptides with cargo into the cytosol occurs after interaction with the inner member bilayer and destabilization of the micelle [32].

### 2.2. Drug Delivery Systems with CPPs

An important obstacle in drug delivery is the lack of the ability of drugs to penetrate into the cells. However, this limitation has been overcome due to the discovery of cell-penetrating peptides such as HIV1-TAT. Furthermore, new DDS that can efficiently use the unique cell-penetrating property of TAT have been created. Kwon and colleagues designed a TAT-asparaginase complex that can be effective against lymphoblastic leukemia, where leukemic cells largely rely on the external supply of asparagine [40]. Their experiments demonstrated the ability of the TAT-asparaginase complex to penetrate both hepatocyte cell line (HeLa) and MOLT-4 tumor cell line with remarkable efficiency, while results of previous investigations reported that TAT domains showed comparable ability to translocate a variety of drugs across the membranes of many cell types. However, such penetrating ability makes TAT-protein complexes very potent therapeutic agents, simultaneously making them more dangerous for normal cells, leading to undesired side effects. To attenuate the extent of possible damage for normal tissues, the authors integrated the ATTEMPTS DDS with TAT-asparaginase complex. The complex was preliminarily inactivated due to the electrostatic interaction between oppositely charged surface functional TAT groups and negatively charged heparin, which completely blocks the penetrating ability of TAT [40]. Once the target is reached, positively charged protamine, which has a higher binding affinity to heparin compared to TAT, is introduced into the circulation [40,41]. Thus, protamine binds to heparin displacing TAT-asparaginase and recovering TAT’s penetrating ability due to which asparaginase can enter the cells. HeLa and MOLT-4 cell lines were also used to examine if the heparin-protamine regulation allows for the controlled release of the TAT-asparaginase. The original ATTEMPTS model also includes a specific antibody for targeting the complex to the tumor cells. However, Kwon and colleagues, instead of using the original antibody directed system, imitated cellular uptake of asparaginase and conducted in vivo experiments by direct injection of asparaginase-encapsulated L5178Y cells into the mice. The difference between the means of survival time of the tumor injected mice and the asparaginase-encapsulated tumor injected group constituted 1.7 days in favor of the latter [40]. In addition, the model of PTD-modified ATTEMPTS has been accomplished and tested in vivo by Shin and colleagues, who used carcinoembryonic antigen monoclonal antibodies for targeting of TAT-Gel (gelonin fusion chimera) to LS174T cells that show high carcinoembryonic antigen expression [42]. The data showed that the tumor growth was inhibited by 65% after protamine injection into the TAT-Gel-heparin pretreated mice.

Wu and colleagues have developed an oral charge reversible DDS for penetration of mucus and epithelial barriers [43]. The system represents poly(lactic-co-glycolic acid) (PLGA) nanoparticles conjugated with octa-arginine peptides (R8) and phosphoserines (Pho) through polyethylene glycol (PEG) links (P-R8-Pho NPs). Mucus penetration is achieved due to the slightly net negative charge (zeta potential −2.4 mV) of the system that avoids electrostatic interactions with negatively-charged mucin [44]. The low net negative charge of the system was, in turn, established due to mutual cancelation of charges between conjugated cationic R8 and anionic phosphoserine moieties. However, to interact with the negative plasma membrane of the epithelial cells and to pass through them, the system needs to have positively charged functional groups, and that is where the charge reversal takes place. The charge reversal occurs due to the cleavage of phosphate moiety on phosphoserine by intestinal alkaline phosphatase that is expressed in the intestinal epithelium [45]. Thereby, overall charge of the system becomes more positive (+7.4 mV) due to arginine-rich R8 peptides acting as cell penetrating agents allowing the system to pass through the epithelial barrier. Thus, the charge change of the system, which is equal to ~9.8 mV, occurs due to the response to the intestinal alkaline phosphatase environment. The system illustrates good mucus penetration ability that was almost as good as that of conventional PEGylated nanoparticles, 14.8 × 10^−6^ cm/s and 12.6 × 10^−6^ cm/s, respectively. Insulin was used as a model drug for in vivo studies with diabetic mice [43]. Insulin, a hormone secreted by pancreatic beta cells, regulates the synthesis of glucose through the secretion of glucagon, transcriptional regulation of major gluconeogenic genes, such as PCK1 and G6PC and activation of signaling pathways responsible for gluconeogenesis, including PI3K and MAPK pathways [46,47]. Oral administration of insulin-loaded P-R8-Pho NPs demonstrated blood glucose reduction by 32% in 3 h, whereas no decrease in glucose levels were achieved by orally administered free insulin when applying the same 50 IU/kg dosage in each case (Table 1). The system also demonstrated 6.0 ± 0.9% of relative bioavailability while free insulin showed only 0.40 ± 0.15% [43]. 

Penetration into cancer cells via R8-conjugated DDSs has also been reported. Delivery of CRISPR/Cas9 complex for pancreatic cancer treatment was designed by Li and col-leagues. The complex was encapsulated into R8-conjugated cationic liposomes [48]. CRISPR-Cas9 complex contains DNA endonuclease Cas9 that cleaves double-stranded DNA of interest and sgRNA that is used as a guide compound. CRISPR-Cas9 mediates an epigenomic correction of mutated cancer genes and is thus now used in various anti-tumor applications, such as stromal-targeting therapies, cancer immunotherapy and oncolytic virotherapy [49]. In addition, R8 peptide-linked DDSs were also examined for delivery of small interference RNAs to treat liver cancer. Small interference RNAs mediate cancer-specific gene silencing through RNA interference, the process where certain regions of targeted messenger RNAs are cleaved [50,51]. He and colleagues reported about ~80% tumor volume inhibition in HepG2 tumor-bearing mice [52]. R8 conjugated DDSs found their application even in wound healing. Li and colleagues demonstrated that R8 mediated delivery of collagen/chitosan gel increased angiogenesis and granulation tissue formation as well as enhanced deposition of collagen and thus, resulted in improved cutaneous wound healing [53].

## 3. Targeted Delivery of Peptides

### 3.1. Molecular Mechanism of Targeted Delivery of Peptides

The idea of targeted delivery of drugs was first coined at the beginning of 20th century by Paul Ehrlich [54]. The concept arose from the fact that diseased tissue has various complex cellular and non-cellular components [55], which might be targeted by drugs that can act as “magic bullets” selectively eliminating diseased cells without damaging healthy ones thereby improving the utilization of drugs and reducing their side effects [56,57]. Since those times, thousands of different DDSs that are capable of side-directed accumulation have been reported, and the concept of targeting was updated and divided into active and passive targeting. In passive targeting, drugs accumulate at diseased sites due to intrinsic characteristics of DDSs such as size, shape and charge, and due to distinctive properties of the targeted sites such as local vasculature and lymphatic drainage. At tumor sites, for example, nearby vasculature is leaky, and lymphatic drainage is impaired or absent [58]. Such conditions exhibit the so-called enhanced permeation and retention effect on DDSs, which allows preferential accumulation of polymers with high molecular weight as well as nanoscale particles of approximately 20–500 nm in diameter within the tumor tissue [59]. Active targeting is, in turn, receptor-directed and achieved by attaching receptor-specific ligands to the drug carrier or drug itself. Peptides being natural ligands for many receptors in our body have found a place among commonly used targeting agents in drug delivery. Most commonly, targeting peptides act as a delivery system for targeting various tumor cells or tissues due to overexpression of tumor-specific markers [60]. There are several molecular target candidates for this group of peptides including integrin receptors, aminopeptidase N, extracellular matrix (ECM) components and EGFRs (Figure 1).

Integrins control growth and survival of tumor cells in the process of tumor cell escape and infiltration into blood or lymphatic vessels by managing different steps of motility and invasion of tumor cells [61]. Integrin receptor family consists of 24 heterodimeric cell-adhesion receptors with a combination of α and β subunits for each receptor [62]. The Arg-Gly-Asp (RGD) peptide can bind to one of four integrin classes called RGD-binding receptors. RGD receptors have eight members (ανβ1, ανβ3, ανβ5, ανβ6, ανβ8, α8β1, α5β1 and αIIbβ3) and among them ανβ3, ανβ5, α5β1 and ανβ6 are known to be involved in progression of cancers and their further metastasis. Moreover, ανβ3 heterodimer is overexpressed in the blood vessels of tumor cells [63]. It was shown that the binding of RGD peptides to integrin receptors can inhibit the expression of glycoproteins of ECM, such as vitronectin and fibrinogen leading to the decreased cell adhesion and tumor formation [64]. Moreover, after interaction with ligands, integrins undergo CvME and CME. This means that targeting peptides with drug conjugates can further penetrate the tumor cell, decreasing toxicity towards healthy cells [65].

Another target for targeting peptides is mammalian aminopeptidase N or CD13, which is overexpressed on most major tumors [66]. It belongs to the family of type II zinc-dependent metalloproteinases and promotes angiogenesis, tumor growth and metastasis in different cancer cells via participation in enzymatic cleavage of the polypeptide chain [67]. A targeting peptide, Asn-Gly-Arg (NGR), can specifically bind to CD13 in tumor blood vessels. Particularly, it was shown that an NGR peptide interacts with the zinc-dependent catalytic site of porcine CD13 via the side chains of Asn and Arg. Additionally, a cyclic structure of NGR peptide tightens the distance between the Asn and porcine CD13 residue forming stable and efficient binding [66]. This high specificity and stability can be used to deliver different tumor inhibiting factors such as tumor necrosis factor and truncated tissue factor, which lead to caspase mediated apoptosis and thrombosis in tumor vasculature, resulting in tumor cell death and tumor infarction [68,69,70]. Furthermore, an NGR peptide can be combined with RGD peptide to target CD13 and ανβ3 integrin receptors to enhance binding affinity and targeting efficiency [71].

After passing through the walls of blood vessels, drugs can be delivered directly to the stroma of diseased tissues by peptides targeting a number of ECM components. The WYRGRL sequence can bind to the type-II collagen (α1 chain) of cartilage tissue, which is one of the main ECM targets in arthritis [72]. A recent study demonstrated that WYRGRL conjugated with dexamethasone (corticosteroid), is effectively retained in the deep zones of cartilage via specific interactions with cartilage-specific collagen type II and increases the efficacy of the drug against osteoarthritis [73]. The FHKHKSPALSPVGGG sequence can selectively bind to another component of ECM in tumor cells, called tenascin-C, which is overexpressed in the tumor microenvironment [72]. More recently, a PL3 peptide also demonstrated specificity against tenascin-C. PL3-coated nanoparticles were accumulated in tenascin-C positive areas in clinical tumor samples suggesting that PL3 peptide can be used as a targeting peptide for selective delivery of therapeutics to the site of solid tumors [74]. Moreover, one peptide sequence can target different ECM components delivering several therapeutics at the same time. It was demonstrated that PPRRGLIKLKTS sequence coated iron oxide nanoworms and metallic silver nanoparticles can recognize overexpressed fibronectin and tenascin-C in glioblastoma and prostate carcinoma xenografts [75].

Finally, EGFR, which is a protein overexpressed on multiple cancer cells, can also be a reliable target for peptides to deliver different therapeutics. There are several targeting peptides (KCCYSL, LTVSPWY, FCDGFYACYMDV and SVDNPHVC) that can bind to human EGFR. Among them, SVDNPHVC sequence or Disruptin, which is derived from the eight amino acid segment of EGFR, were reported to specifically bind to EGFR, preventing EGF-dependent EGFR dimerization that resulted in subsequent EGFR degradation in cancer cell lines [76]. More recently, four analogs of targeting peptide GE11 (YHWYGYTPQNVI) were reported to reach a high uptake level by triple negative breast cancer cell line with high expression levels of EGFR [77]. Furthermore, an engineered EGFR targeting (with GE11 peptide) self-assembly amphiphilic peptide nanoparticle (GENP) was used to deliver gemcitabine and the poly-ADP-ribose polymerase (PARP) inhibitors Olaparib to treat BRCA mutant pancreatic cancer. GENP-Gem-Ola initiated single stranded break of DNA and inhibited PARP-mediated DNA repair leading to cell apoptosis of BRCA2 mutant capan-1 cells in in vitro and in vivo experiments [78]. 

### 3.2. DDSs with Targeting Peptides

A prominent sequence of targeting peptides is the RGD tripeptide (Figure 1). The tripeptide binds several types of integrin receptors called ανβ3 and ανβ5 whose elevated expression was observed in the endothelium of newborn vessels of solid tumors [79]. A great variety of RGD peptide alterations have been developed up until this point, which include but are not limited to such prominent examples such as RGD-4C, and cyclic iRGD, cRGDyK, cRGDfC, cRGDfK and cN-Me-VRGDf [80]. Prolongation of the sequence with appropriate amino-acids and introduction of cyclization are made to improve stability of the peptide against proteolytic degradation and enhance its affinity to the integrin receptors [81]. According to Zhou and colleagues, RGD-4C (ACDCRGDCFCG), which is cyclized by two disulfides bonds between C2-C10 and C4-C8, has a 200-fold higher affinity to αvβ3 or αvβ5 than linear peptides [82]. But abundant research with unmodified RGD sequences also showed comparably good results and a targeting ability of the tripeptide. The studies of delivery of vanadium carbide quantum dots by exosomes performed by Cao and colleagues are examples of RGD-conjugated DDS. Comparison of the RGD-conjugated systems with non-conjugated exosomes was conducted using Fluorescence spectroscopy indicating that RGD improved the targeting since higher accumulation of the DDSs in tumor sites and lower accumulation in the liver, kidney, spleen and heart was achieved [83]. Wang and colleagues, who have fabricated RGD integrated red blood cell-based multimodal probe (RBCp) for fluorescence imaging-guided tumor surgery and photodynamic therapy, could achieve a 2.1-fold higher photoacoustic signal at the tumor sites of mice treated with the RGD-conjugated system than those treated with the system without RGD. It was ascribed that RBCps with RGD illustrated ~2 times higher tumor-to-liver ratio of the system accumulation than RBCps without RGD [84]. 

iRGD-conjugated PLGA/lipid nanoparticles were developed for delivery of indocyanine green (ICG, 775 Da) and tirapazamine (TPZ, 178 Da) to the tumor sites. iRGD has a dual role of a guiding and penetrating agent [85]. ICG, in turn, is a photosensitizer that produces reactive oxygen species (ROS) in response to NIR irradiation under oxygen-available conditions, while in the hypoxic tumor environment, ICG is capable of enhancing the cell-killing properties of the TPZ, which destroys cells by means of radical addition of hydrogen from macromolecules in vicinity. Conjugation of iRGD with nanoparticles provides penetration up to 90 µm into the 4T1 tumor spheroid, which corresponds to 12 cell layers. Targeting properties of the system were measured in 4T1 tumor-bearing mice. The system exhibited decreased localization in the liver and increased accumulation in the tumor. However, more nanoparticles were observed in other major organs (heart, lung, kidney, spleen) compared to the free ICG. Weights of the tumors 13 days after treatment constituted ~0.1 g and ~1.4 g for the ICG/TPZ-loaded iNPs and control saline solution, respectively [85].

Overexpression of RGD motif-specific integrins can be observed not only at tumor sites. Tian and colleagues detected a strong increase in the expression of avb3 integrins in the vessels of ischemic regions. The investigators achieved strong suppression of inflammatory response and cellular apoptosis in the sites of lesions of ischemic brain of mice due to the use of curcumin-loaded cyclic RGDyK conjugated exosome (cRGD-Exo-cur). Curcumin (368 Da), also known as diferuloylmethane, is a member of the polyphenol family, extracted from Curcuma longa [86]. The drug possesses antipathogenic, anti-inflammatory and antioxidant abilities and thus is used for the treatment of various disorders, including chronic inflammation, neurodegenerative diseases, metabolic syndrome, liver disease and cancer [87]. The group also examined how conjugation of the exosomes with the cRGD peptide affects the targeting of the systems to the ischemic regions in the mice brains. The results demonstrated an ipsilateral/contralateral ratio of 19 for cRGD-Exo-cur localization in the brain of the tested animal. However, although the increase of signal intensity was higher in the sites of lesions, the fluorescence signal intensity was also enhanced in other organs such as the liver and lungs. According to the literature, the increased localization of the drug in the liver was due to a high expression of αvβ3 integrins, while in the case of the lungs, it happened because of the larger size of cRGD-Exo in comparison with unmodified exosomes [88]. 

Another type of tripeptides that are used for targeting are NGR peptides. NGRs are ligands for aminopeptidase N (CD13) receptors that are also highly expressed in angiogenic tumor vasculature [89]. Yan and colleagues have developed a nanoscale DDS containing three different peptides called PMI, BIM and iNGR clustered together with lanthanide nanoparticles (LDC-PMI-BIM-iNGR). PMI and BIM, having pro-apoptotic properties, act as therapeutics, while cyclic iNGR functions as a guiding agent [90]. The constructed system showed a longer than 48 h. In vitro cell viability studies showed a cytotoxicity of ~75% after 3 days incubation of HCT116 cell lines with LDC-PMI-BIM-iNGR. Measurements of apoptosis levels in the same cell lines demonstrated 97% of dead cells after 72 h incubation, while incubation with conventional Nutlin-3 resulted in the death of 93% of cells. According to in vivo fluorescence studies with HCT116 tumor-bearing mice, LDC-PMI-BIM-iNGR revealed a strong signal at the tumor site while low signal was detected in the liver and kidney, and background signal was detected in the brain, heart, lung and spleen. The system accounted for 90% of growth inhibition in the xenograft tumor 12 days after treatment with 2.5 mg/kg dosage, which is superior to the results shown by doxorubicin (DOX) [90]. 

## 4. Stimuli-Responsive Peptides

Authors stimuli-responsiveness is the ability of DDSs to alter the configuration in response to certain triggers. It is an important property of smart DDSs that allows them to function specifically and controllably in order to reduce the potency of adverse effects and enhance the therapeutic efficacy of drugs. Different stimulation agents (pH, light, magnetic field, enzymes) can significantly change the properties of DDSs, modulating their cell membrane permeability, internalization, size shrinkage and drug release [91]. Stimuli-responsive systems can be triggered manually by photothermal, magnetic, electric or ultrasonic impacts externally or in response to local environmental factors such as pH, temperature, redox state and concentration of some molecules (e.g., O_2_, urea, enzymes) [92,93]. 

### 4.1. Enzyme-Responsive Peptides

Among environment-responsive peptides, enzyme-responsive peptides are most frequently reported, and enzymes that are chosen to act as appear to be peptidases (or proteases/or proteinases). Peptidases are enzymes that belong to the class of hydrolases, which break covalent peptide bonds (>C(=O)NH-R) down using water [94]. A wide variety of peptidases have been reported to accumulate more frequently at places of lesion such as tumor sites and ischemic regions. The classes of those peptidases can be divided into metallo- (e.g., gelatinases, matrilysins), cysteine- (e.g., cathepsin B, cathepsin C), serine- (e.g., uPA, PSA, thrombin), threonine- (e.g., testes-specific protease 50, threonine aspartase 1) and aspartic proteases (e.g., cathepsin D, cathepsin E, memapsin) [95]. Correspondingly, researchers have become interested in using such features of tumor tissues to design biocompatible peptidase-responsive DDSs. 

Pancreatic ductal adenocarcinoma (PDAC) currently accounts for 25% of deaths from cancer in the U.S. and as the data suggests this number will increase to 50% by 2030. Treatment is challenging, partially due to its resistance to chemotherapy and immunotherapy [96]. Gemcitabine (263 Da) is a deoxycytidine nucleoside analog and is used as a standard treatment choice against metastatic PDAC [97]. It mediates its anti-proliferative function via the blockage of cell cycle progression at the G1/S-phase boundary [98]. The use of gemcitabine improves survival for approximately 2–3 months, however this is almost invariably accompanied by the acquisition of chemo-resistance [99]. The use of smart DDSs is one of the approaches that improves drug efficiency. There is a large number of various peptidase-responsive peptides used in DDSs, but a select few have received particular attention due to their outstanding efficiency (Figure 1). One of such peptides is GFLG (Gly-Phe-Leu-Gly) tetrapeptide that can be cleaved by cathepsin B, a cysteine protease, which has higher levels of expression in most types of tumors in comparison to normal tissue [100]. Zhang and colleagues have reported on the usage of GFLG in a DDS. The system, which they designed, represents PEGylated lysine dendrimer nanoparticles conjugated with gemcitabine through cathepsin B-cleavable GFLG [101]. Being constructed from the amino acid residue, the dendrimer possesses a high level of biodegradability and water-solubility. PEGylation, in turn, improves solubility of the system and decreases immunogenicity, while together with the branched architecture of the dendrimer, allows the drug to have a longer blood circulation time due to which less frequent dosing may be applied. The DDS is designed to release gemcitabine as a result of GFLG cleavage by cathepsin B. The studies have shown more than an 80% higher gemcitabine release in the cathepsin B environment (in vitro) compared to the control environment without it. Overall, the nanoparticles showed relative tumor suppression volume of 82 ± 38% in a 4T1 murine breast cancer model, having no signs of cytotoxicity to normal cells, which proves great biocompatibility of the system and the efficiency of GFLG particularly. According to the drug release kinetic, 60% of loaded gemcitabine was released within 30 min, while 90% of the drug was released in 24 h (Table 2) [101]. 

Tumor environment-responsiveness of GFLG was also utilized by Jiang and colleagues who used DOX as a drug of interest, which has only 5% bioavailability after oral administration [102]. DOX is the most well-known anthracycline antibiotic that has been successfully used as an anti-cancer drug to treat hematological and solid tumor malignancies for more than 40 years [103]. The anti-tumor properties of DOX are due to the generation of ROS, inhibition of topoisomerase II and intercalation into DNA, leading to the disruption of gene expression [104]. The response rate for metastatic lesions when using DOX is 25%–40% [105]. However, there are still a number of limitations that restrict its usage, including chemo-resistance, irreversible cardiotoxicity and reversible nephrotoxicity. Packing DOX into specific cargos were shown to mitigate some challenges and reduce the toxicity of the drug. There are now a number of liposomal vehicles that have been approved by the U.S. FDA. Those liposomal nanocarriers, however, cannot eliminate tumor drug resistance [106]. In his study, Jiang and colleagues used the peptide to link copper sulfide nanoparticles with DOX to examine the efficiency of the system against lymphoma. Intracellular in vitro accumulation studies showed almost two-fold higher relative fluorescence intensities for CuS-GFLG-DOX in comparison to free DOX. The proposed DDS also generated an enhanced apoptosis rate in Raji cell lines compared to the control [102]. Wang and colleagues also used a GFLG-conjugated DDS to deliver DOX. According to apoptosis studies on a multidrug resistant breast cancer cell model, MCF-7/ADR, the proposed mPEGylated dendron–GFLG–DOX demonstrated 2.6 higher apoptotic rate than free DOX constituting [107].

Daunomycin, which is also a member of the anthracycline family and an alternative chemotherapeutics to DOX, has a number of side effects. The drug, which was first isolated from *Streptomyces peucetius*, is used for acute lymphoblastic or myeloblastic leukemia treatment [110]. The antitumor effect of the drug is mediated by various mechanisms, including topoisomerase II poison, generation of ROS, DNA impairment and dissociation of H1.1 linker histones from DNA leading to the higher-order chromatin structure destruction [111]. Dokus and colleagues have proposed a DDS for delivery of GFLG-conjugated daunomycin to the pancreatic adenocarcinoma sites. In vivo studies on PANC-1 tumor-bearing mice resulted in a decrease in cell viability [109]. 

There are also some examples of conjugating GFLG to a DDS for photodynamic therapy (PDT). PDT is an alternative cancer treatment that uses molecules activated by light of appropriate wavelength to generate ROS in targeted cells and thus selectively kill tumor cells [112]. Wang and colleagues could achieve a higher than 80% tumor inhibition rate of the triple negative breast cancer model due to linking of squaraine photosensitizers to ultrasmall superparamagnetic iron oxide via GFLG [108]. Guaraine photosensitizers are the class of dyes used in photodynamic therapy (PDT). 

Among all types of proteases, the group of matrix metalloproteinases (MMPs) is the largest one, which has been proved to have an essential role in the development of malignant tumors [113]. Taking this fact into consideration, various DDSs that respond to a MMP-rich environment have been developed. PLGLAG hexapeptide has been frequently reported as a stimuli-responsive component of those DDSs. One of the novel PLGLAG-conjugated DDSs was reported by Han and colleagues who proposed an interestingly designed polysaccharide-based self-shrinking DDS [114]. The system is composed of poly(amidoamine) (PAMAM) dendrimer conjugated with hyaluronic acid (HA) via an MMP-2-cleavable PLGLAG peptide link. HA, which is a ligand for CD44 receptors that is overexpressed in many cancer types, is used as a targeting agent of the system. So-called shrinking of the system occurs due to the splitting of HA moieties from the central drug-containing PAMAM under the influence of MMP-2. The idea of the system is to improve accumulation of the drug near the tumor vasculature due to larger size particles (~200 nm), and then trigger their shrinking in response to the tumor environment releasing small particles (~10 nm) that are more penetrative. The tumor inhibition rate for the DOX loaded system in A549 tumor-bearing mice equaled 62.5%, which was higher than that of free DOX (50.2%). The advantage of the system is preferable biodistribution showing almost no toxic effect in major organs including the liver [114]. Since DDSs, which include enzyme-responsive peptides, are designed in a way that the cleavage of one linker peptide releases one molecule of a drug, a number of peptide cleavages can be used as release kinetics data. Cleavability of PLGLAG in the MMP-2 environment, which was examined by Wang and colleagues, demonstrated that 54% of the linkers were cleaved in 8 h [115]. Being the main enzymes responsible for ECM degradation, MMPs also play a role in the development of many other pathologies that involve excessive ECM degradation including arthritis, aortic aneurysms, periodontitis and many others [116]. Additionally, there were reports about MMP-2/9 overexpression after myocardial infarction which resulted in the death of cardiomyocytes [117]. This could mean that MMPs-responsive peptides could be used as a stimuli-responsive part for various potential DDSs to treat numerous diseases. 

Peptidase-responsive DDSs can also be conjugated with targeting agents for a more accurate localization of drug cargoes. Nosrati and colleagues designed a layer of glycine coating implemented on iron-based magnetic nanoparticles to improve biocompatibility of the system, concurrently allowing proteinase K-dependent release of the conjugated methotrexate under lysosomal conditions [118]. Mimicking the structure of folic acid, methotrexate not only possesses the ability to target folate receptors, which are overexpressed on the surfaces of many cancer cells, but also demonstrates anticancer therapeutic properties [119]. Glutamic acid residue in the methotrexate is able to be conjugated to glycine on the surface of the nanoparticles through the formation of a peptide bond that can be cleaved by proteinase K expressed in lysosomes. Kinetic studies illustrate that the solution containing proteinase K resulted in a 70% methotrexate release in 12 h, whereas under conditions without the enzyme, only 40% of the drug was released during the same period of time. Cytotoxicity data showed no significant difference between cell viability of control HFF-2 and HEK-293 cell lines and the ones incubated with glycine-coated or bare nanoparticles, which proves that the system is highly biocompatible. Moreover, cell viability of MCF-7 breast cancer cell lines decreased by ~50% when a 1.6 µM solution of methotrexate-conjugated glycine-coated nanoparticles was applied to it. At the same concentration, free methotrexate showed ~70% cell viability [118]. 

### 4.2. pH-Responsive Peptides

The ability to be cleaved by proteases is not the only property of peptides that can be used to design stimuli-responsive DDSs. Being made of charged constituents, peptides can also respond to pH changes. Such property can be especially useful for DDSs since there are a number of pathological conditions such as ischemia, arthritis, atherosclerosis and tumor that are known to exhibit decreased pH at the sites of lesion [120]. Among various reported pH-sensitive peptides, a family of pH-low insertion peptides (pHLIP) stands out due to its ability to penetrate through the membrane [121]. The mechanism of action of pHLIPs is now well-understood: at physiological pH, pHLIP has coiled conformation and is negatively charged and at this hydrophilic state, it cannot penetrate the plasma membrane. However, in an acidic environment, C terminus and negative residues of pHLIP get protonated, resulting in a more neutral, lipophilic state and a change of conformation from a coiled to alpha helix, providing penetration through the plasma membrane via neutral C terminus and anchoring to it as a transmembrane protein [122,123].

Huang and colleagues described pH- and thermoresponsive gold nanocages (pPGNCs), of which pH-sensitive properties are attributed to a 36 amino acid long pHLIP peptide [124]. In turn, poly(di(ethylene glycol) methyl ether methacrylate-co-oligo(ethylene glycol) methyl methacrylate) (PEGDMA-MMA) is attributed for thermosensitivity that responds to the NIR irradiation-triggered increase in temperature. The temperature-induced collapse of the gel opens the pores of the nanocages allowing release of the loaded drug molecules. DOX-loaded pPGNCs demonstrated almost two times lower accumulation in heart and ~5 times higher accumulation in tumor than free DOX. However, the system also exhibited two times higher accumulation of the drug in the liver compared to the free DOX. It was shown that at the pH 6.5, significantly more (one-and-a-half times more for MCF-7 cells and four times more for Adriamycin-resistant MCF-7 cells) DOX was internalized into the tumor cells than at the pH 7.2, confirming the pH-responsive cell-penetrating abilities of the pHLIP peptide. The in vivo anticancer studies on drug-resistant MCF-7 tumor-bearing mice showed 97% tumor growth inhibition by DOX-loaded pPGNCs under the light irradiation (Table 3) [124]. 91% tumor growth inhibition has been achieved in 30 days of treatment by Han and colleagues who designed the delivery of gemcitabine by 35 amino acid long pHLIP-conjugated iron-based nanoparticles [125]. In addition, the possibility of delivering peptide nucleic acids of sizes as large as 7 kDa via 38 amino acid long pHLIP membrane-penetrative abilities was reported [126]. Nucleic acids cause changes in signaling pathways of cancer cells via the synthesis of functional proteins and the degradation of mRNA through RNA interference [127]. Thus, this approach could potentially expand the spectra of applicable drugs in chemotherapy [126].

## 5. Peptide-Based Self-Assembly Scaffolds

Production of biomaterials fabricated via self-assembling of short peptides or their derivatives is a promising approach for creating novel therapeutics in regenerative medicine. The extraordinary signaling capability and therapeutic effectiveness of peptide scaffolds have been confirmed in animal models. There are quite a few classes of self-assembling peptide-based scaffolds of peptide amphiphiles, Fmoc-di and tri peptides, self-complementary ionic peptides, hairpin peptides, etc. Self-assembly is an entropy driven process that appears in nature by folding a polypeptide sequence to form tertiary structures. They can be designed for implementing bioactive signaling strategies and improving cell signaling capabilities (growth factors, RNA, DNA, etc.) [128]. Moreover, manipulations with small building blocks provide an opportunity for development of more sophisticated and hierarchical structures based on peptides with given properties and a modulated degree of decomposition. An important approach nowadays is the use of machine learning for designing peptide sequences with given properties. Amphiphilic peptides are able to self-assemble to nanoarchitectures that contain hydrophobic and hydrophilic domains. It may have two, three, or four blocks providing new structural and functional properties and affinities for communication with cellular membranes or intracellular organelles. Amphiphilic peptide scaffolds were made using the human nuclear Ki-67 protein, which acts as a biosurfactant and provides a steric and electrostatic charge obstacle against the collapse of mitotic chromosomes [129].

There are a number of triggers that can induce a simultaneous self-assembly process, the most common is change in pH that leads to the shift of equilibrium from charged to uncharged groups resulting in dominance of hydrophobic interactions. A simple pH adjustment with alkali or acid does not lead to generation of acceptable homogeneous scaffolds, and therefore transglutaminage, urease or gamma gluconolacton are usually utilized. A macroporous scaffold was prepared by using a self-assembly process at various conditions including subzero temperatures. FmocPhePhe based scaffolds with a pore size of 50–150 µm are composed of tightly packed nanofibers that can be used for mammalian cell cultivation and for loading with the hydrophobic drugs [130]. Another advantage of using peptides for scaffold generation is the ability to utilize a concentration below 1%, which is economically advantageous. Wakabayashi and colleagues studied the self-assembly of 9-fluorenylmethoxycarbonyl (Fmoc)-(Leu)n-Gln-Gly to nanofibers. After the in-situ hydrogel preparation, the enhanced green fluorescent protein was fused with MRHKGS tag, which was attached to the functional groups of fibers using enzymatic reaction catalyzed by transglutaminase. This approach illustrates the option of scaffold modification with covalently attached signaling molecules or growth factors [131]. Pandit and colleagues studied the mechanism of self-assembly of tetrapeptide Boc-Trp-Leu-Trp-Leu-OMe to spherical nano-/microspheres upon dissolution in ethanol. Peptide strands hydrogen-bonded to form anti-parallel β sheets, in which the tryptophan ring pointed toward one side of the β sheet and the leucine side chains pointed toward the other side. Remarkably, the nanospheres can be obtained at the 0.02 mM concentration and it is quite unusual that it did not involve the aromatic p-p stacking of tryptophan rings and was mediated by the hydrophobic effect. The advantage of this system is that due to a low (0.02 mM) concentration, nanocarriers did not aggregate in contrast to what was observed at 0.156 mM. Curcumin was immobilized with the efficiency of release using these nanospheres [132]. 

Fatouros and colleagues designed and studied lipid-like peptides, such as ac-A6K-CONH2 and ac-A6D-COOH, to mimic natural lipids having a hydrophilic head and a hydrophobic domain. Critical concentrations of micelle formation for lipid-like peptides in phosphate buffer saline were 0.12 mg/mL for A6K-CONH2, 0.09 mg/mL for KA6-CONH2, 0.08 mg/mL for ac-A6D-COOH and 0.06 mg/mL for DA6-COOH, which are low enough to demonstrate perspective from an economic point of view. These carriers do not affect Caco-2 cell proliferation in comparison with the control. A slow Nile red release by ac-A6D-COOH and ac-A6K-CONH2 nano-vesicles was detected with reaching substantial levels after 6 and 4 h, respectively [133]. Another approach is oxidation triggered self-assembly when DPEIM peptide was conjugated with hexapeptide EIMIME, designed by Song and colleagues. Peptide containing moiety under hydrogen peroxide oxidation triggered self-assembly, co-assembling the peptide with covalently linked photosensitizer or drug, which resulted in spherical particle formation with a size of 38 nm, having a combinatorial therapeutic effect. ROS, which is induced by the photosensitizer, instantaneously provides the photodynamic therapy. Efficiency of scaffolds was demonstrated by administering them to 4T1 breast tumor-bearing mice. The treatment with DPEIM led to a decline in the growth of the tumor tissues in comparison with free camptothecin, demonstrating the improved therapeutic outcome attributed to the peptide vehicles [134]. Cui with colleagues studied peptide Nap-Gly-Phe-Phe-Lys-His instant self-assembly to microfibers at pH ≈6.0, which is close to the pH micro environment of the human skin. Then, a fibroblast growth factor (FGF-2) was incorporated into fibrous alginate with antibiotic-loaded peptide hydrogel Nap-Gly-Phe-Phe-Lys-His. The dual DDS has a potential for wound healing due to a good mechanical property and appropriate release kinetic, where an antimicrobial agent can be quickly released from the peptide scaffold, while the FGF-2 was gradually delivered within one week [135].

Another interesting approach is the use of multidomain peptide (MDP) scaffolds, which are made of sequence KSLSLSLRGSLSLSLKGRGDS (termed SLac) and also have the ability for self-assembling via anti-parallel stacking of peptides into fibrillar ribbons. Monocyte chemotactic protein (MCP) has a smaller mass (7.6 kDa) and hydrodynamic volume (9.14 nm^3^) in comparison with IL-4 that is larger (14.0 kDa) with a volume of 16.9 nm^3^. The difference in the charge at physiological pH is also important, for example, MCP has +5 and IL-4 +7.9, to establish the particular differences in Mw and hydrodynamic volume, that are milestone factors for estimation diffusivity of heavy charged biomolecules via a nanofibrillar hydrogel. This biomaterial with MCP illustrated the largest number of infiltrating cells and the greatest level of infiltration at 3 days compared to SLac alone or IL-4 loaded scaffolds. At a later stage, scaffolds with absorbed IL-4, or MCP-1 and IL-4 revealed several distinct blood vessels with circulating red blood cells [136,137].

Zhang and colleagues investigated an amino acid sequence, in which self-assembly is triggered by ionic strength into β-sheet positively charged nanofibers within full pH range (pH 0–14). It forms nanofibrils and then hydrogel at physiological condition at a minimum concentration, which is meaningfully lower when compared to the previously published results. The hydrogel can be obtained even at dense conjugation with a bioactive OVA257-264 peptide, an in vitro study of which illustrated significantly enhanced CD8+ T cell activation [138]. Luo and colleagues fabricated scaffolds using d-EAK16 sequence that contains d-amino acids Ac-(Ala-Glu-Ala-Glu-Ala-Lys-Ala-Lys)2-CONH2. The self-assembly process to a secondary β-sheet structure was triggered by an increase in temperature [139]. The self-assembly process was mediated by inherited factors as the primary structure and chiral induction as well as by external factors such as concentration, temperature, ionic strength, pH change and hydrolyzing agents, and therefore, can be called a smart nanomaterial. It is known that peptides of D-amino acids are resistant to biodegradation by natural enzymes and for this reason these noncovalent scaffolds are more durable. Moreover, modified peptides utilizing the chiral amino acids can provide scaffolds with given properties. Enzymes and growth factors can be adsorbed/incorporated to the nanofibers without usage of additional cross-linking agents and to form biological gradients.

A prospective approach of drug formulation preparation for intravenous administration is the application of Gemini natural amino acid-based surfactants due to the lack of side effects of the surfactant product degradation as well as other beneficial effects such as biocompatibility, antimicrobial activity etc. Potential applications of Gemini peptides as non-viral delivery system agents are discussed in the review. The Gemini surfactants can contain positively charged (Arg, Lys), neutral (Ser, Ala, Sar) and negatively charged (Asp) amino acids, and thiol containing (Cys) as polar head groups, which overall gives peptides the ability of self-assembly in aqueous solutions to micelles [140]. As was previously shown, the fate of metabolites of drug and supplementary substances is important during treatment. Jin and colleagues focused on establishing the effect of metabolites of Gemini surfactants, which can be utilized for gene delivery. They compared various parameters of several surfactant compositions such as unsubstituted (16-3-16), substituted with pyridinium head groups (16(Py)-S-2-S-16(Py)) or with a glycynyl-lysine di-peptide (16-7N(GK)-16). The metabolism of these substances was found to be dissimilar as 16-3-16 was stable in PAM 212 cells, whereas 16(Py)-S-2-S-16(Py) was metabolized via oxidation and dealkylation, producing metabolites with high toxicity. The 16-7N(GK)-16 was digested primarily via methylation, acetylation, glucose conjugation, palmityl and stearyl conjugation. One can conclude that the establishment of the metabolism of Gemini surfactants offers insight for future guidelines in the design and improvement of effective Gemini surfactants with better biocompatibility [141].

Gemini surfactant-like peptides contain prolines that are responsible for turn-forming units (Ser-Val-Ala-Ala-Leu-Ala-Ala-Gln-Gly-Leu-Leu-Asn-Ala-Pro-Lys) (APK). The peptide exists in linear conformation as a Gemini surfactant. Peng and colleagues compared a single-tailed surfactant-like peptide A6K and APK, where APK has a significantly stronger ability to undergo self-assembly and to encapsulate hydrophobic compounds (paclitaxel, doxorubicin, etomidate and propofol). The tested formulations revealed anti-tumor or anesthetic efficiency comparable to their respective clinical formulations. It is worth mentioning the advantage of this DDS; APK has inhibiting activity against the growth of various strains of *E. coli, S. aureus and C. albicans*. Etomidate and propofol formulations, which were included into the APK assembled carriers, illustrated strong antimicrobial activity [142]. In another study, cationic lipodipeptides were investigated against photogenic planktonic and biofilm culture stains (*Enterococcus faecium, Staphylococcus aureus, Klebsiella pneumoniae, Acinetobacter baumannii, Pseudomonas aeruginosa, and Enterobacter* spp.) and *Candida species* [143]. The authors demonstrated that the branching and shortening of the fatty acid chain length and concurrent attachment of a hydrophobic N-terminal amino acid create a substance with an enhanced affinity. One approach was when arginine-rich Gemini cationic surfactants were linked with cystine diamide and lysine amide linkers as spacers. Antimicrobial activity of the surfactant in the 10% normal human serum was in the range of 64 and 256 µg/mL and had a critical micelles concentration in the range of 0.3–7.5 mM. As expected, the critical aggregation concentration was directly proportional and interrelated with hydrophobicity. It was stated that Gemini surfactants were selective antimicrobial agents with rather low hemolytic and cytotoxic activities. The surfactant with the cystine diamide spacer indicated lower cytotoxicity compared to lysine amide counterparts, however it illustrated lesser antibiofilm and antimicrobial activities in serum. Some branched fatty acid chains and N-terminal hydrophobic amino acid residues revealed higher selectivity to pathogens compared to human cells [143].

Quaternary ammonium Gemini surfactants with enhanced nucleic acid complexation ability exhibited a relatively low cytotoxicity. Twenty-two novel peptide-modified Gemini surfactants with different alkyl tails and peptide spacer modifications were tested for their potential for gene delivery. Dendrimer-like Gemini surfactants have critical micelle concentration in the range of 45–107 µM. The authors tried to establish the structure activity relationship of the peptide-modified Gemini surfactants and to determine the fundamental conformational requirements compulsory for the gene delivery systems [144]. According to the in vitro tests, the highest transfection efficacy and minimal cytotoxicity were attributed to the Gly-Lys modified Gemini surfactants possessing the hexadecyl tail, C16-7N(Gly-Lys)-C16. This system illustrated an eight-time increase in secreted protein in PAM 212 with 20% increase in cell viability relative to the 1st-generation unsubstituted Gemini surfactants. The authors reported that an increase in the size of the attached peptides led to a decrease in the transfection effectiveness and cell viability. In contrast, the incorporation of a hydrocarbon linker into the peptide chain reduced the transfection efficacy of substances with dipeptides [144]. A balance between the hydrophilic and hydrophobic characteristics of the compound is necessary since it results in physicochemical parameters conducive to the gene delivery process using intravenous administration. Cardoso and colleagues investigated conventional and serine-derived bis-quat Gemini surfactants in order to efficiently mediate gene delivery to mitochondria in HeLa cells and to promote gene expression, or in combination with the enhancer lipids DOPE and cholesterol. It was observed that the transfection of up to 40% of the cells was twice of what was attained with Lipofectamine 2000 (25%). The authors demonstrated that a high yield of mtDNA delivery to the organelle was reflected by the replication of 104 copies of mtGFP mRNA per a plated cell and by significant GFP production, resulting in an increase of green fluorescence of the cells. The most promising DDS, showing a combination of high transfection efficacy and low cytotoxicity, were characterized physicochemically to establish a structure activity relation. These complexes are 14−2−14/mpDNA, 14−2−14/mpDNA/HL, (14Ser)2N5/mpDNA/HL and (16Ser)2N5/mpDNA, and have a size from 0.274 to 0.8 µm and possess various surface charges from positive to negative. (14Ser)2N5/mpDNA/HL, exhibited negative zeta potential, preventing undesired interactions with serum proteins [145]. Therefore, one can conclude that application of Gemini surfactants is a very promising approach for commercialization due to a simple method for synthesis, inexpensive components, biocompatibility and low toxicity of metabolites.

QSAR and SAR are widely used for the prediction of biological activity of drugs. However, a quite novel approach is the use of bioinformatics and the calculation of descriptors such as the autocovariance value of peptides that facilitate the determination of the optimum drug-carrier pair. Hardy and colleagues utilized four descriptors: constitutional (Mw), electronic (number of hydrogen bond donors and number of hydrogen bond acceptors) and physicochemical (xLogP). Calculation of interactions between 18 peptides and drugs (acyclovir, amphotericin B, cryptolepine, doxorubicin, 5-fluorouracil (5FU), isoniazid, resveratrol, curcumin, paclitaxel and indomethacin) is discussed [146]. Along with various well known and widely used cycodextrines, another supramolecular structure, calixarene, is able to form host–guest inclusion complexes and can be used for formulation of DDS. In a recent study, in-silico multilevel docking investigation was carried out to enhance the pharmacological profile of the second-generation tyrosine kinase inhibitors with drugs (dasatinib, lapatinib and nilotinib) via formation of host–guest inclusion complexes with calixarene. The binding with calix[n]arenes (n = 4, 5, 6 and 8) can be modulated by introducing additional groups (-SO3H, tert-Butyl, iso-Propyl, -COOH, -C2H4OH and -C2H4NH2). It was established that the driving forces of inclusion complex formation are steric fit, π effects, intra/intermolecular hydrogen bonding and electronic effects leading to 1:1 stoichiometry drug—inclusion complex [147]. It is well known that albumins have a great affinity to form a complex with various drugs including peptide structure, and this phenomenon can be utilized for designing DDS. Metwally and colleagues studied simulation of interactions of albumin and gelatin nanoparticles with resveratrol and curcumin. By applying approaches of chemoinformatics molecular dynamics and molecular docking, it was possible to determine the optimum carrier for each of the chemopreventive agents [148]. Moreover, Casalini introduced the term “computational microscope”, which provides simulations at atomic scale that facilitate the understanding of the impact of molecular interactions on crucial factors such as kinetic of release rate and the response of DDS to external environment, giving insights that are challenging or impossible to acquire using experimental setup due to the complexity of the system. This novel paradigm in nanomedicine underlined the significance of in-silica tools to study the interactions between peptide or protein based nanoparticles with drugs [149]. Macromolecular modeling of the interaction between the drug and DDS carrier has high expectations due to the development of novel software and the creation of more powerful supercomputers, as well as the utilization of sources of artificial intelligence.

## 6. Ongoing/Recently Completed Clinical Trials

As discussed above, peptide-based DDSs demonstrated their efficacy against various cancer diseases in preclinical studies. Currently, there are several clinical trials that are investigating the therapeutic effects of different peptide drug conjugates, including ANG1005, CBX-12, melflufen and bicycle peptides (BT5528 and BT8009).

An open-label, multicenter phase II study was conducted to test the efficacy, safety and tolerability of ANG1005, a new taxane derivative consisting of three paclitaxel molecules covalently linked to a 19-amino acid Angiopep-2 peptide, in 72 adult patients with measurable recurrent brain metastases from breast cancer (BCBM), with or without leptomeningeal carcinomatosis. An angiopep-2 peptide was designed to cross the blood–brain barrier (BBB) through interaction with the LRP1 transport system. Benefit of the treatment (stable disease or better) was seen in 77% of patients intracranially and 86% of patients extracranially. Moreover, in leptomeningeal carcinomatosis, intracranial disease control reached 79% of the patients with an estimated median overall survival of 8.0 months (95% CI, 5.4–9.4) (NCT02048059) [150]. These results confirmed that ANG1005 can penetrate BBB and deliver paclitaxel to the central nervous system to exhibit its antitumor activity. Currently, an open-label phase III (ANGLeD) study was designed, but is not yet recruiting, to evaluate the effectiveness of ANG1005 in patient survival compared to a Physician Best Choice control in 150 HER2-negative breast cancer patients with newly diagnosed leptomeningeal disease and previously treated brain metastases (NCT03613181).

CBX-12 is a pH-Low Insertion Peptide (pHLIP) based platform conjugated to a topoisomerase inhibitor, exatecan, which inhibits the topoisomerase enzymes preventing relieve of DNA supercoiling after its replication, transcription and chromatin remodeling [151]. At the same time, pHLIP also specifically targets the low pH environment of the tumor in an antigen-independent manner allowing the insertion of the peptide into the cancer cell membrane and subsequent release of the agent into the tumor cell through glutathione reduction of the linker [152]. Cybrexa Therapeutics is now recruiting for a phase I/II open-label, multicenter, dose-escalation, safety study of CBX-12 on 112 patients with advanced or metastatic refractory solid tumors. Primary outcome measures for the study are to evaluate the incidence of treatment-emergent adverse events, recommended dose and overall response rate (NCT04902872).

Melflufen is a novel peptide-drug conjugate that rapidly and selectively releases alkylating agents into tumor cells by targeting aminopeptidases. A phase II HORIZON trial evaluated the efficacy of melflufen conjugated to dexamethasone in 157 patients with relapsed and refractory multiple myeloma (RRMM). The overall response rate to melflufen was 29% in the all-treated population (triple-class-refractory disease, extramedullary disease and refractory to previous alkylator therapy populations), with 26% in the triple-class-refractory population. In the all-treated population, the average response time to the therapy was 5.5 months, while average overall survival was 11.6 months at a median follow-up of 14 months with manageable adverse effects (NCT02963493) [153]. A larger randomized, controlled, open-label, phase III OCEAN study was announced to test the efficacy and safety of melflufen plus dexamethasone versus pomalidomide plus dexamethasone on 495 patients with RRMM that is refractory to lenalidomide. The primary endpoint for this study is progression-free survival, while key secondary endpoints include overall response rate, duration of response and overall survival (NCT03151811) [154]. However, despite its accelerated approval, the U.S. FDA is now requiring the manufacturer to suspend enrollment in the OCEAN trial due to an increased risk of death from the therapy.

Bicycles are small (1.5 kDa) synthetic, structurally constrained peptides 9–20 amino acids long with three cysteine residues within their sequence. These cysteine residues maintain the peptide in a rigid conformation by reacting with a small molecule linker [20]. Two clinical trials (BT5528-100 and BT8009-100) were designed to determine the anti-tumor effect of novel bicycle peptides developed by Bicycle Therapeutics. A phase I/II multi-center, open-label BT5528-100 trial is recruiting 166 patients with advanced solid tumors associated with Ephrin type-A receptor 2 (EphA2) expression. BT5528 is a Bicycle Toxin Conjugate (BTC), consisting of a bicyclic peptide targeting this tumor antigen EphA2 overexpressed in different tumors such as non-small-cell lung cancer (NSCLC), ovarian cancer, triple-negative breast cancer (TNBC), gastric/upper gastrointestinal (GI), pancreatic and urothelial cancers, and linked to a cytotoxin (monomethyl auristatin E [MMAE]) through a tumor microenvironment cleavable Val-Cit linker. Phase I is a dose escalation study primarily designed to evaluate the tolerability and safety of BT5528 alone, or in combination with, antitumor monoclonal antibody, nivolumab, as well as to determine a recommended dose for phase II. Primary outcome measures include the evaluation of safety, adverse events, progression-free and overall survival rate (NCT04180371) [155]. Another phase I/II, multicenter, first-in-human, open-label dose-escalation BT8009-100 study is also recruiting 146 patients with Nectin-4 expressing advanced solid tumors malignancies. BT8009 is another BTC in which a Nectin-4 binding bicyclic peptide is conjugated via an inert sarcosine spacer chain, and a cleavable linker, to the same toxin MMAE as BT5528. Overexpression Nectin-4 has been reported in various tumors (bladder, breast, esophageal, colorectal, lung, ovarian and pancreatic cancers), therefore it represents an efficient tumor binding target [156]. The main goal of this study is to determine a safe recommended dosage, side effects and effectiveness of BT8009 alone, or in combination with, nivolumab (NCT04561362). Table 4 summarizes the ongoing clinical trials. Overall, it seems that peptide drug delivery systems based on Angiopep-2 peptide, pHLIP, melflufen and bicycles can be further implemented into clinical trials and may demonstrate an efficient approach to targeted delivery of drugs to tumor sites with potential to develop into novel methods of treatment.

## 7. Conclusions

Advances in DDSs in the scope of application of peptide conjugates has been achieved in the delivery strategies and therapeutic index of drugs. In actuality, many presently formulated anticancer medications have unfavorable pharmacodynamic and pharmacokinetic properties, concurrently with several restrictions on the dosage regime and side effects in the conventional dosage form. The importance was shown of the chemical reactivity of a cleavable linker that directly depends on the lysosomal pH for efficient substance release, i.e., the acid-cleavable N-acyl hydrazine linker, enzyme hydrolyzed bonds, or non-cleavable peptide bond linkers that release the drug after metabolism. Recent advances in peptide-based drug delivery have allowed for the engineering of new stimuli-responsive peptide-based carriers (pH, near infrared light, magnetic field, enzymes proteases, etc.) for the treatment of various diseases. DDSs can undergo cyclic size shrinkage, PEG linker detachment, recurrence of ligand targeting and reactive drug delivery. A number of research papers illustrate advances of the peptide-based systems carriers in terms of a great potential for targeted delivery of chemotherapy and gene interference. DDSs with CPPs overcome important obstacles of drug penetration through the cells. Arginine-rich R8 peptides illustrated excellent results of CRISPR/Cas9 complex delivery for pancreatic cancer with poor outcome. Various targeting peptides were compared, including Asn-Gly-Arg, which have a specific affinity for binding with CD13 on tumor blood vessels. Moreover, several examples of GENP designed for delivery of gemcitabine and the PARP inhibitors aimed for treatment of mutant pancreatic cancer were discussed. A comparative analysis of Gly-Phe-Leu-Gly conjugated with an antitumor drug DDS showed promising results and prospective application. In this review, we did not cover the area of peptide conjugated vaccines and antibody–drug conjugates, as it is a large and quite specific topic. 

The commercialization of novel peptide conjugated DDSs and innovative formulations is expected to have a potential positive impact on society via accelerated recovery of patients by increasing therapeutic efficiency and reducing side effects. The use of in silico approaches for the prediction of solubility, zeta potential and confirmation of pep-tide-drug conjugates will stimulate the discovery of more advanced DDSs. In spite of the promising nonclinical studies of DDSs, the implementation of additional clinical trials or development of hybrid peptide-based carriers is necessary to improve the translation of developed innovative peptide-based DDSs for efficient application in chemotherapy or growth factor delivery for regenerative medicine.

## Figures and Tables

**Table 1:** R8 peptide conjugated DDSs.

Delivery Vehicle	Therapeutic Agent	Mechanism of Action of the Therapeutic Agent	Application	Outcome	References
PLGA nanoparticles	Insulin	Secretion of glucagon, transcriptional regulation of gluconeogenic genes	Diabetes mellitus treatment	Blood glucose reduction by 32% in three h	[43]
Cationic liposomes	CRISPR/Cas9	Correction of mutated tumor genes	Pancreatic cancer treatment	~70% tumor volume suppression	[48]
Upconversion nano-onions	Small interference RNA	Gene silencing through the cleavage of messenger RNAs	Liver cancer treatment	~80% tumor volume inhibition	[52]
Collagen/chitosan gel	Collagen/chitosan	Increased angiogenesis, collagen deposition, granulation tissue formation	Wound healing	98 ± 4.7% surface healing after 2 weeks	[53]

**Table 2:** GFLG conjugated drug delivery systems.

Delivery Vehicle/Targeting Agent	Drug	Mechanism of Action of the Drug	Cancer Type	Outcome	References
PEGylated lysine dendrimer nanoparticles	Gemcitabine	Antiproliferative properties through the blockage of cell cycle progression	Breast cancer	89.9% tumor growth inhibition	[101]
Copper sulfide nanoparticles	DOX	Generation of ROS, inhibition of topoisomerase II, disruption of gene expression	Lymphoma	49.6 ± 1.3% apoptosis rate	[102]
mPEGylated dendron	DOX	Generation of ROS, inhibition of topoisomerase II, disruption of gene expression	Breast cancer	31% apoptotic rate	[107]
CREKA (Cys–Arg–Glu–Lys–Ala) peptide Fibronectin–targeting	Squaraine photosensitizer	Induction of cytotoxicity through the generation of ROS	Triple negative breast cancer	83.5 ± 8.7% tumor inhibition rate	[108]
SKAAKN (Cys–Lys–Ala–Ala–Lys–Asn) peptide	Daunomycin	Topoisomerase II poison, generation of ROS, DNA impairment	Pancreatic ductal adenocarcinoma	0.1 ± 0.1% cell viability	[109]

**Table 3:** pHLIP conjugated DDSs.

Delivery Vehicle/Targeting Agent	Drug	Mechanism of Action of the Drug	Cancer Type	Outcome	References
Gold nanocages	DOX	Generation of ROS, inhibition of topoisomerase II, disruption of gene expression.	Breast cancer	97% tumor growth inhibition	[124]
PEGylated Fe_3_O_4_ nanoparticles	Gemcitabine	Antiproliferative properties through the blockage of cell cycle progression	Pancreatic ductal adenocarcinoma	91.2% tumor growth inhibition after 30 days of treatment	[125]
38 amino acid long	Peptide nucleic acids	Synthesis of functional proteins and the degradation of mRNA through RNA interference	Melanoma	7 kDa peptide translocation	[126]

**Table 4:** Ongoing/recently completed clinical trials.

#	Study Title	Disease	Treatment (Intervention)	Estimated Enrollment	Current Status and Phase	Trial Number
1	ANG1005 in Breast Cancer Patients With Recurrent Brain Metastases	Breast Cancer, Brain Metastases	Participants intravenously received ANG1005 up to a maximum of one year, or until disease progression or adverse events	72 participants	Completed, Phase II	NCT02048059
2	ANG1005 in Leptomeningeal Disease From Breast Cancer (ANGLeD)	Leptomeningeal Carcinomatosis, Leptomeningeal Metastases, Brain Metastases, HER2–negative Breast Cancer	Participants intravenously received ANG1005 or active comparator: Physician’s Best Choice (capecitabine or eribulin or high-dose intravenous (IV) methotrexate)	150 participants	Not yet recruiting, Phase III	NCT03613181
3	Study of CBX–12 in Subjects With Advanced or Metastatic Refractory Solid Tumors	Solid Tumor Adult, Epithelial Ovarian Cancer, Small Cell Lung Carcinoma	CBX–12 administered on a daily × 5 every 3 weeks schedule or a daily × 3 every 3 weeks schedule in ovarian and small lung cancer cohorts	112 participants	Recruiting, Phase I/II	NCT04902872
4	A Study of Melphalan Flufenamide (Melflufen) in Combination With Dexamethasone in Relapsed Refractory Multiple Myeloma Patients (HORIZON)	Multiple Myeloma	Patients received intravenously 40 mg of melflufen on day 1 of each 28-day cycle and once weekly oral 40 mg of dexamethasone (20 mg in patients older than 75 years)	157 participants	Not yet recruiting, Phase II	NCT02963493
5	A Study of Melphalan Flufenamide (Melflufen)–Dex or Pomalidomide–Dex for RRMM Patients Refractory to Lenalidomide (OCEAN)	Multiple Myeloma	Patients received intravenously 40 mg of melflufen on day 1 of each 28-day cycle and once weekly oral 40 mg of dexamethasone or Pomalidomide 4 mg orally daily on days 1 to 21 and dexamethasone 40 mg once weekly of each 28-day cycle	495 participants	Active, not recruiting, Phase III	NCT03151811
6	Study BT5528–100 in Patients With Advanced Solid Tumors Associated With EphA2 Expression	Advanced Solid Tumor Identified as Positive for EphA2 Tumor Expression by Central Laboratory (Phase I), Non Small Cell Lung Cancer Identified as Positive for EphA2 Tumor Expression by Central Laboratory (Phase II)	Patients receive intravenous infusion of BT5528 once a week alone or with nivolumab on a 4-week cycle at the selected dose	166 participants	Recruiting, Phase I/II	NCT04180371
7	Study BT8009–100 in Subjects With Nectin–4 Expressing Advanced Solid Tumors Malignancies	Advanced Solid Tumor, Urinary Bladder Neoplasm, Pancreatic Neoplasms, Triple Negative Breast Neoplasms, Carcinoma, Non-Small-Cell Lung, Stomach Neoplasm, Esophageal Neoplasms, Ovarian Neoplasm	Patients receive intravenous infusion of BT8009 once weekly alone or with nivolumab on a 4-week cycle at the selected dose	146 participants	Recruiting, Phase I/II	NCT04561362
